# Anatomical and Functional Effects of Oral Administration of Curcuma Longa and Boswellia Serrata Combination in Patients with Treatment-Naïve Diabetic Macular Edema

**DOI:** 10.3390/jcm11154451

**Published:** 2022-07-30

**Authors:** Olimpia Guarino, Claudio Iovino, Valentina Di Iorio, Andrea Rosolia, Irene Schiavetti, Michele Lanza, Francesca Simonelli

**Affiliations:** 1 Eye Clinic, Multidisciplinary Department of Medical, Surgical and Dental Sciences, University of Campania Luigi Vanvitelli, 80131 Naples, Italy; olimpia.guarino@hotmail.it (O.G.); valentina.diiorio@unicampania.it (V.D.I.); dr.rosolia@gmail.com (A.R.); mic.lanza@gmail.com (M.L.); francesca.simonelli@unicampania.it (F.S.); 2 Department of Health Sciences, University of Genoa, 16132 Genoa, Italy; irene.schiavetti@unige.it

**Keywords:** Boswellia serrata, curcumin, diabetic macular edema

## Abstract

Anti-vascular endothelial growth factor nowdays represents the standard of care for diabetic macular edema (DME). Nevertheless, the burden of injections worldwide has created tremendous stress on the healthcare system during the COVID-19 pandemic. The aim of this study was to investigate the effects of the oral administration of Curcuma longa and Boswellia serrata (Retimix^®^) in patients with non-proliferative diabetic retinopathy (DR) and treatment-naïve DME < 400 μm, managed during the COVID-19 pandemic. In this retrospective study, patients were enrolled and divided into two groups, one undergoing observation (Group A, n 12) and one receiving one sachet a day of Retimix^®^ (Group B, n 49). Best-corrected visual acuity (BCVA) and central macular thickness (CMT) measured by spectral-domain optical coherence tomography were performed at baseline, then at one and six months. A mixed-design ANOVA was calculated to determine whether the change in CMT and BCVA over time differed according to the consumption of Retimix^®^. The interaction between time and treatment was significant, with F (1.032, 102.168) = 14.416; η^2^ = 0.127; *p* < 0.001, indicating that the change in terms of CMT and BCVA over time among groups was significantly different. In conclusion, our results show the efficacy of Curcuma longa and Boswellia serrata in patients with non-proliferative DR and treatment-naïve DME in maintaining baseline CMT and BCVA values over time.

## 1. Introduction

Diabetic retinopathy (DR) is one of the main causes of working-age visual loss in industrialized countries. It is a long-term manifestation of diabetic microangiopathy which most commonly affects the eyes, the peripheral nerves, and the kidneys [1]. DR is caused by damage to the retinal blood vessels that affects the macular region and the peripheral retina, resulting in an overall reduction of visual function [1].

Diabetic macular edema (DME) is the result of intraretinal fluid accumulation in extracellular location, due to the breakdown of the blood-retinal barrier [2]. This process is caused by the release of pro-inflammatory substances. Hyperglycemia stimulates a hyper-activation of microglia with the consequent development of the inflammatory process mediated by interleukin (IL)-1β, tumor necrosis factor (TNF)-α, IL-6, and vascular endothelial growth factor (VEGF) [3]. In concomitance, the alteration of ion exchanges between photoreceptors and Müller cells creates a fluid overflow with the formation of intracellular edema. The production of VEGF molecules contributes to increased vascular permeability and thus vascular homeostasis loss [4].

DME formation can occur in both the proliferative and non-proliferative forms of DR and its onset is typically associated with some characteristic symptoms, including visual blurring and distorted vision. Fluorescein angiography (FA), through the detection of macular capillary hyperpermeability, and optical coherence tomography (OCT), through the detection of intra and subretinal fluid, represent the specific diagnostic investigations currently used to detect DME [5,6].

According to the current literature, when DME is considered subclinical for its size and localization and is associated with a good visual acuity, the patient can be monitored over time with no treatment administered [7,8].

There are some natural substances, not considered to be medications, that have been shown to help in the treatment of systemic and ocular pathological conditions [9,10,11]. Among these, the root of Curcuma longa, rich in polyphenols, is a potent anti-inflammatory agent and prevents the formation of reactive oxygen species. The latter can lead to pathological processes, like cell apoptosis, angiogenesis, and inflammation ending in retinal pathologies [12].

Boswellic acids derived from the gum of the Boswellia serrata (a plant native to India) also have anti-inflammatory and anti-arthritic activities [13,14]. Recent studies have shown that the association of active ingredients derived from Curcuma longa and Boswellia serrata acts synergistically to counteract the pathways of inflammation at multiple levels [15,16].

Retimix^®^, a combination of the two described substances, would allow for the exploitation of the combined and synergistic activity of its components in the control of the inflammatory processes occurring in retinal disorders, including DR.

On this background, the aim of this study was to investigate the anatomical and functional effects of the oral administration of Curcuma longa and Boswellia serrata in patients with non-proliferative DR and treatment-naïve DME, managed during the COVID-19 pandemic.

## 2. Methods

In this study, patients with treatment-naïve DME managed during the COVID-19 pandemic were retrospectively evaluated at the Retina Unit of the University of Campania “Luigi Vanvitelli”. Institutional review board approval was obtained for a retrospective consecutive chart review by the Vanvitelli University Ethics Committee. The study adhered to the guidelines of the Health Insurance Portability and Accountability Act and was performed in accordance with the tenets of the Declaration of Helsinki.

Inclusion criteria were: patients with type 2 diabetes treated indifferently with antidiabetic therapy based on metformin or insulin, having non-proliferative DR with DME and central macular thickness (CMT) < 400 μm. Diagnosis of DR and DME was based on patients’ history and multimodal imaging evaluation including fundus color picture, FA and spectral-domain (SD)-OCT. All patients were treatment-naïve and were enrolled during the COVID-19 pandemic under public health restrictions, with limitations in terms of operating rooms available and daily scheduled visits.

The exclusion criteria were: the presence of any other retinal disease or ocular disorder that could be associated with the development of macular edema (e.g., recent history of cataract and/or vitreoretinal surgery in the previous 6 months), hyperopia or myopia > 6 diopters, and any other concomitant nutritional supplements therapy. Additionally, patients with media opacities that could influence image quality were also excluded from the study.

Subjects who met all inclusion criteria were enrolled in the study and divided in two groups, one undergoing observation (Group A) and one receiving Retimix^®^ (Group B). A detailed systemic and ocular history was obtained and patients underwent a complete ocular examination at each visit, including Best-Corrected Visual Acuity (BCVA) testing using 4-m ETDRS charts, slit-lamp biomicroscopy, intraocular pressure evaluation with Goldmann applanation tonometry, and CMT measurement by SD-OCT (Cirrus 4000, Carl Zeiss Meditec, Dublin, CA, USA). The overall treatment duration was 6 months and data were collected at baseline (T_0_), 1 month (T_1_), and 6 months (T_2_). All OCT scans were acquired with follow-up function.

Group B patients received one sachet a day of Retimix^®^ formulation which contains Casperiva^®^, EyePharma, corresponding to demethoxycurcumin and bisdemethoxycurcumin plus Boswellic acid in phosphatidylcholine phytosome for a total of 0.5 g phospholipidic-complex; one single foil pouch of powder per day.

All patients were also followed by a diabetologist, to ascertain a good metabolic control.

Anatomical and functional changes, in terms of CMT reduction and BCVA improvement, were evaluated over time and compared between the two groups. The percentage of patients having systemic hypertension and dyslipidemia were also recorded.

### Statistical Methods

Continuous variables are summarized as mean with standard, and categorical data are expressed with frequency and percentage.

A mixed-design ANOVA was calculated to determine whether the change of CMT and BCVA over time (from baseline to 1 month and 6 months) differed according to the consumption of Retimix^®^ formulation. In particular, the model included time as a within-subject factor, and sex, CMT, and treatment group as a between-subject factor. Age and BCVA at baseline were included in the analysis as covariates.

For data which violated the normal distribution, *p*-values were adjusted using the Greenhouse–Geisser correction, and the adjusted *p*-values were reported.

Alpha for statistical test was set at 0.05.

## 3. Results

Sixty-one (61) patients, 31 females (50.8%) and 30 males (49.2%) with a mean age of 64.2 (±14.13) years old, were enrolled and divided in two groups: observation (Group A, n = 12) and treatment (Group B, n = 49).

All baseline demographic and clinical ocular and systemic characteristics of the total cohort are summarized in Table 1.

There were no statistically significant differences in the two groups regarding demographics, ocular (BCVA, CMT, pseudophakia), and systemic parameters (systemic hypertension and dyslipidemia) at baseline evaluation. No patients received either pars plana vitrectomy or retinal laser treatments before the inclusion or during the study.

The mixed-model ANOVA showed that time alone had a non-significant main effect: the CMT at the end of the follow-ups was not significantly different in the two groups from that at the beginning of the study in the total cohort, F (1.032,102.168) = 0.107; ƞ^2^ = 0.001 (*p* = 0.75).

Likewise, the main effect of group on the size of CMT (regardless of the time) was not significant, F (1,99) = 3.862; ƞ^2^ = 0.038; *p* = 0.052.

Conversely, the interaction between time and treatment was significant, with F (1.032,102.168) = 14.416; ƞ^2^ = 0.127 (*p* < 0.001), indicating that the change in CMT among groups was significantly different (Table 2).

**Figure 1:**
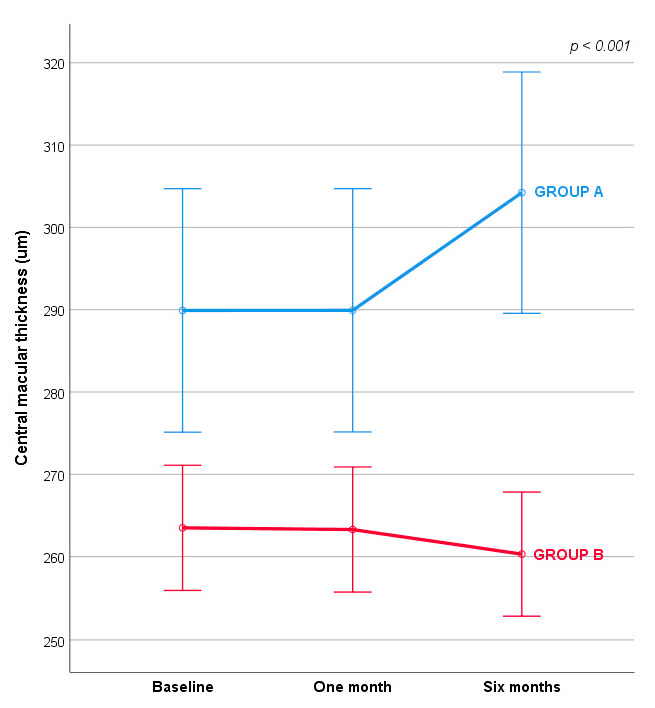
Central macular thickness changes over time. Covariates appearing in the model are evaluated at the following values: Age, years = 63.697, T_0_ Visus = 53.377. Error bars: +/−1 SE.

Specifically, there was no overall natural change in CMT over time, but there was a significant reduction of CMT in patients of Group B at six months (Figure 1).

After the first month of treatment both groups remained mostly stable. After six months there was a significant difference between the groups: Group B in particular remained unchanged compared to Group A, which showed a worsening in CMT dimension.

The interaction between time and treatment on BCVA was also significant, F (1.084, 108.386) = 12.514; ƞ^2^ = 0.111; *p* < 0.001, indicating that the change in BCVA among groups was significantly different (Table 3).

## 4. Discussion

DME is the most prevalent vision-threatening complication of DR, particularly among adults with type 2 diabetes [17]. Although anti-VEFG nowadays represents the standard of care, the burden of injections worldwide has posed a tremendous stress to the healthcare system.

During the COVID-19 pandemic, the postponement of appointments and treatments in non-monocular patients with DME was proposed [18].

Recently, data from the DRCR Protocol V randomized clinical trial suggest that it is safe to observe patients with centre-involved DME and good vision (20/25 or better). Overall, a total of 702 patients were managed with either laser, aflibercept, or observation, and at 2 years the mean BCVA was 20/20 in all three cohorts [7].

In a subanalysis of the RESTORE study, patients were stratified by baseline central retinal thickness (CRT < 300 μm, 300–400 μm, and >400 μm). Among patients treated with ranibizumab greater gains in BCVA were achieved in patients with a higher baseline CRT [19].

Similarly, results from the Protocol I study, suggest that ranibizumab treated patients with DME with higher baseline central subfield thickness (CST; ≥400 μm) achieved greater visual gains.

On this background, the aim of our study was to investigate the anatomical and functional effects of the oral administration of Curcuma longa and Boswellia serrata in patients with treatment-naïve DME, managed during COVID-19 pandemic.

Public restrictions limited the number of intravitreal injections performed and the number of visits for all patients including diabetic patients. Following the published guidelines for intravitreal injections during the pandemic [18], we postponed non-urgent cases and decided to treat with anti-VEGF injections only patients having DME > 400 μm. Our results showed that patients receiving one sachet of oral Retimix^®^ did not show a significant change in CMT at six months when compared to patients undergoing observation.

The specific characteristics of the Retimix^®^ active ingredients, combined with the actions described above, make them an ideal agent as a preventive treatment in many pathologies due to inflammatory and vascular factors, as for DR.

The Casperiva (Retimix^®^) formula consists of curcuminoids, the main one being curcumin, together with demethoxycurcumin and bisdemethoxycurcumin. Among the active ingredients extracted for the formulation there are also several boswellic acids, belonging to the triterpenoid family; AKBA (3-Acetyl-11-keto-beta-Boswellic Acid) is the most documented and active [15].

Recent studies have shown that curcumin is implicated in the functions of natural responses to inflammation, both with a direct action on metabolic pathways, and on the enzymes expression level, transcription factors, and cytokines, through the suppression of the activation of the nuclear inflammatory transcription factor NF-kB, which regulates the expression of the genes of pro-inflammatory cytokines (IL-1, IL-6, TNFα), and secondly “downregulates” the expression of COX-2 (cyclooxygenase-2) [20].

One of the action mechanisms of the substance is its ability to induce peroxisome proliferator-activetedreceptor gamma (PPAR-γ) activation. PPARs play an important role in lipid degeneration, immune regulation, the control of reactive oxygen species (ROS) and VEGF, matrix metalloproteinases-9 (MMP-9), and docosahexaenoic acid (DHA). In addition, PPAR gamma is expressed in RPE cells.

Boswellic acids such as 11-keto-β-boswellic acid (KBA) and its acetylated counterpart (AKBA) have been proposed as selective inhibitors of 5-lipoxygenase (5-LO) because they regulate the inflammatory response function through the inhibition of leukotrienes. Boswellic acids have an action on 5-LO, inhibiting leukotrienes, which increase vascular permeability, as well as mast cells and histamine release and neutrophil recall [21]. In particular, AKBA has been shown to have a proven direct inhibitory action on VEGF.

The action of curcuminoids on VEGF, therefore, is indirect, because it passes through PPAR-γ, whereas AKBA has a direct action on VEGF expression. This allows a dual action both on VEGF and neo angiogenesis, and on the inflammation control to which the tissue is subjected, with a control of the inflammatory process at multiple levels [22].

The limits of the bioavailability of natural extracts, and therefore of their therapeutic efficacy, have been overcome thanks to the patented Phytosome^®^ technology. This technology encloses the active ingredients in a new phospholipidic complex (phosphaditilserine and phosphaditilcholine) developed by Eye Pharma SpA—Genova, Italy, in collaboration with Indena SpA—Milan, Italy that protects them from gastric degradation with a complete absorption in the intestine [23,24,25]. All these molecular characteristics of these active ingredients built the rationale for us to use Retimix^®^ in the management of treatment-naïve diabetic patients with DME who could not receive intravitreal injections or laser treatment due to COVID-19 pandemic restrictions. The main limitations of our study include its retrospective nature and the relatively small number of patients included. Nevertheless, this was not an impediment for the statistical analysis.

In conclusion, our results suggest the protective role of the oral administration of Curcuma longa and Boswellia serrata in patients with non-proliferative DR and treatment-naïve DME in maintaining baseline CMT and BCVA values over time. Considering its anti-inflammatory and anti-angiogenic properties, the Retimix^®^ formulation could be also considered as an adjuvant therapy for patients with DME receiving intravitreal injections, but this awaits further prospective validation.

## Figures and Tables

**Table 1:** Baseline demographic and clinical ocular and systemic characteristics of study patients.

		Total N = 61	Group A N = 12	Group B N = 49	*p*
**Age (years)**		64.2 ± 14.13	65.8 ± 17.76	63.8 ± 13.23	0.44
**Sex**	**Female**	31(50.8)	7 (58.3)	24 (49.0)	0.56
	**Male**	30 (49.2)	5 (41.7)	25 (51.0)	
**CMT (µm) at baseline**		276.3 ± 72.80	291.6 ± 47.63	272.6 (77.67)	0.09
**BCVA (ETDRS Letters)**		52.9 ± 14.60	51.7 ± 18.78	53.2 (13.61)	0.82
**Systemic** **hypertension**		32 (52.5)	7 (58.3)	25 (51.0)	0.65
**Dyslipidemia**		8 (13.1)	2 (16.7)	6 (12.2)	0.68
**Pseudophakia**		18 (29.5)	3 (25.0)	15 (30.6)	0.70

BCVA = best-corrected visual acuity; CMT = central macular thickness.

**Table 2:** Central macular thickness changes over time.

	Baseline	One Month	Six Months	Mixed-Model ANOVA
**Group A**	289.91 (14.79)	289.92 (14.78)	394.22 (14.66)	F (1.032,102.168) = 14.416; ƞ^2^ = 0.127; *p* < 0.001
**Group B**	263.50 (7.61)	263.89 (7.60)	260.30 (7.54)	

Results are expressed as estimated marginal mean with standard error. Covariates appearing in the model are evaluated at the following values: Age, years = 63.697, Visus at baseline = 53.377.

**Table 3:** Best-corrected visual acuity changes over time.

	Baseline	One Month	Six Months	Mixed-Model ANOVA
Group A	53.14 (3.60)	53.14 (3.59)	50.50 (3.69)	F (1.084, 108.386) = 12.514; ƞ^2^ = 0.111; *p* < 0.001
Group B	53.75 (1.85)	53.70 (1.85)	54.44 (1.90)	

Results are expressed as estimated marginal mean with standard error. Covariates appearing in the model are evaluated at the following values: Age, years = 63.697.

## Data Availability

None.
